# Cell wall channels of *Rhodococcus* species: identification and characterization of the cell wall channels of *Rhodococcus corynebacteroides and Rhodococcus ruber*

**DOI:** 10.1007/s00249-022-01599-9

**Published:** 2022-05-14

**Authors:** Claudio Piselli, Lorraine Benier, Cornelia Koy, Michael O. Glocker, Roland Benz

**Affiliations:** 1 Jacobs University Bremen, Campusring 1, 28759 Bremen, Germany; 2 Proteome Center Rostock, University Medicine Rostock, 18059 Rostock, Germany

**Keywords:** Cell wall channel, Channel formation, Octamer, *Rhodococcus corynebacteroides*, *Rhodococcus ruber*, Lipid bilayer membrane

## Abstract

The cell wall of *Rhodococcus corynebacteroides* formerly known as *Nocardia corynebacteroides* contains cell wall channels that are responsible for the cell wall permeability of this bacterium. Based on partial sequencing of the polypeptide subunits and a BLAST search, we identified one polypeptide of *R. corynebacteroides* (PorARc) and two polypeptides (PorARr and PorBRr) from the closely related bacterium *Rhodococcus ruber.* The corresponding genes, *porARc* (606 bp), *porARr* (702 bp), and *porBRr* (540 bp) are constituents of the known genome of *R. corynebacteroides* DSM-20151 and *R. ruber* DSM-43338, respectively. *porARr* and *porBRr* of *R. ruber* are possibly forming a common operon coding for the polypeptide subunits of the cell wall channel. The genes coding for PorARc and for PorARr and PorBRr without signal peptide were separately expressed in the porin-deficient *Escherichia coli* BL21DE3Omp8 strain and the proteins were purified to homogeneity. All proteins were checked for channel formation in lipid bilayers. PorARc formed channels with characteristics that were very similar to those of a previous study. The proteins PorARr and PorBRr expressed in *E. coli* could alone create channels in lipid bilayer membranes, despite the possibility that the two corresponding genes form a porin operon and that both subunits possibly form the cell wall channels in vivo. Based on amino acid sequence comparison of a variety of proteins forming cell wall channels in bacteria of the suborder *Corynebacterineae,* it seems very likely that PorARc, PorARr, and PorBRr are members of a huge family of proteins (PF09203) that form MspA-like cell wall channels.

**Supplementary Information:**

The online version contains supplementary material available at 10.1007/s00249-022-01599-9.

## Introduction

Gram-positive bacteria of the genus *Rhodococcus* belong together with the genus *Nocardia* to the family *Nocardiaceae* (Nouioui et al. 2018). The bacteria are obligate aerobic, mycolate-containing, non-motile and non-spore-forming (Tsukamura 1974; Goodfellow and Alderson 1977; Bell et al. 1998). Besides pathogenic bacteria, such as *Rhodococcus equi* (a pathogen of foals) or *Rhodococcus fascians* (a plant pathogen), the actinomycete genus *Rhodococcus* mostly comprises bacteria found in the soil, in groundwater, sediments and animal dung (Finnerty 1992; Bell et al. 1998; Majidzadeh and Fatahi-Bafghi 2018). Accordingly, many *Rhodococcus* species have distinctive metabolic abilities, which means that they can live on environmental pollutants, or they can transform molecules with interesting properties (Bell et al. 1998; Elsayed et al. 2017). Their morphology can range from coccoid to bacillary, depending on species and specimen type. Colonies are salmon-pink to red colored and teardrop shaped or coalescent mucoid (Bell et al. 1998; Conville and Witebski 2011; Yamshchikov et al. 2010). The genus *Rhodococcus* contains more than 50 named species (https://lpsn.dsmz.de/genus/rhodococcus). The most common pathogenic species in the genus is *Rhodococcus equi*, which causes pulmonary abscesses in foals and possibly also in immunocompromised patients (Yamshchikov et al. 2010; Majidzadeh and Fatahi-Bafghi 2018; Lin et al. 2019). So far, 30 cases of *R. equi* infection in transplant patients have been described, and the clinical diagnosis in 24 of these 30 cases was pneumonia or a lung abscess (Yamshchikov et al. 2010; Lin et al. 2019). Besides *R. equi* infections, cases with *Rhodococcus corynebacterioides/Rhodococcus kroppenstedtii* causing neonatal bacteremia and oligoarthritis (Khalil et al. 2019) and *Rhodococcus kroppenstedtii* causing peritoneal dialysis-associated peritonitis have been reported (Kang et al. 2021).

Gram-positive bacteria of the order *Corynebacteriales* [i.e., the *Corynebacteria–Mycobacteria–Nocardia* (CMN) group] contain a mycolic acid layer surrounding the bacteria that has a similar function as the outer membrane (OM) of Gram-negative bacteria (Brennan and Nikaido 1995; Bansal-Mutalik and Nikaido 2014). Besides the mycolic acids covalently linked to the arabinogalactan–peptidoglycan complex, the OM of the *Corynebacteriales* contains also extractable lipids, in particular glycolipids containing trehalose (Sutcliffe 1998; Sutcliffe et al. 2010). The length of the side chains of the mycolates varies considerably within the order *Corynebacteriales* (Goodfellow et al. 1998). Short mycolic acids (22–38 carbons) were especially found in *Corynebacterium* species, whereas they are with 30–54 carbons medium-sized in *Rhodococcus* (Goodfellow et al. 1998; Bansal-Mutalik and Nikaido 2011). *Mycobacteria* have the longest mycolic acids with 60–90 carbon atoms (Daffé et al. 1990; Teramoto et al. 2015).

There exists emerging knowledge that the mycolic acid layers of bacteria of the order *Corynebacteriales* contain pores in function similar to those in the OM of Gram-negative bacteria (Trias et al. 1992; Trias and Benz 1993; Benz 1994, 2001; Nikaido 2003). This means that the OM of mycolic acid containing bacteria acts as a molecular filter for hydrophilic solutes (Trias and Benz 1993; Lichtinger et al. 1998; Riess et al. 1998). Whereas β-barrel cylinders form all pores in the OM of Gram-negative bacteria, the architecture of cell wall channels varies with the length of the pore-forming polypeptides (Benz 1994; Lichtinger et al. 1998; Costa-Riu et al. 2003; Barth et al. 2010; Abdali et al. 2018). Pores in the cell wall of *Corynebacterium* species are formed from small polypeptides transported in part by a not yet identified mechanism to the OM (Costa-Riu et al. 2003; Barth et al. 2010; Abdali et al. 2018). They contain mostly α-helical structures in the thin mycolic acid layer of the *Corynebacteria* (Ziegler et al. 2008; Barth et al. 2010; Abdali et al. 2013). Pores in the thicker mycolic acid layer of *Mycobacteria*, *Nocardia* and *Tsukamorella* are all formed from oligomers (octamers) of polypeptides with a molecular mass around 20 kDa that are identical or similar in sequence or mass to the subunit MspA of the *Mycobacterium smegmatis* cell wall channel (Niederweis et al. 1999; Riess et al. 2001; Dörner et al. 2004; Kläckta et al. 2011). Octamers of MspA form a goblet-like structure with one channel where the MspA monomer contributes two beta strands to the octameric beta barrel with nonpolar outer surfaces around the pore (Faller et al. 2004). The channel is highly cation selective because of a ring of negatively charged amino acids localized at the opening of the channels towards the cell (Niederweis et al. 1999; Faller et al. 2004). It represents a unique structure that has been suggested for the use in DNA sequencing (Manrao et al. 2012).

In this study, we purified the cell wall channel of *Rhodococcus corynebacteroides* (formerly *Nocardia corynebacteroides* (Yassin and Schaal 2005)) to homogeneity following an established procedure (Riess and Benz 2000). The channels formed by the pure protein had the same conductance as those observed previously (Riess and Benz 2000). The pure protein was subjected to Edman-degradation and sequencing starting from the N-terminal end. The partial sequence was used for BLAST search for subunits of the cell wall channel and allowed the identification of a polypeptide subunit, termed PorARc, which has 41% sequence identity to MspA of *M. smegmatis*. BLAST search for the next homolog of the partial sequence of the *R. corynebacteroides* cell wall channel allowed the identification of two primary polypeptide sequences from *Rhodococcus ruber*. These polypeptides had 30.3 and 31.4% sequence identity to MspA of *M. smegmatis* and presumably either together or alone form the subunits of the cell wall channel of *R. ruber*. All proteins were cloned without signal peptide, expressed in *Escherichia coli* and purified to homogeneity. We studied their properties in reconstitution experiments with lipid bilayer membranes. The channels formed by PorARc and the two subunits of *R. ruber* showed similar characteristics to channels formed by MspA of *M. smegmatis* and to cell wall channels formed by the channel subunits of *Nocardia farcinica* (Niederweis et al. 1999; Kläckta et al. 2011).

## Materials and methods

### Bacterial strains, plasmids, and growth conditions

*Rhodococcus corynebacteroides* DSM-20151 (formerly *N. corynebacteroides* ATCC 14898) and *Rhodococcus ruber* DSM-43338 obtained from DSMZ were grown in 500 mL Erlenmeyer flasks containing 250 mL Corynebacterium-media (Media 53/ DSMZ) at 30 ± 1 °C using a New Brunswick shaker at 120 rpm for 1–2 days. The cells were harvested by centrifugation at 12,000 rpm in a Beckmann J2-21M/E centrifuge for 10 min at 4 °C. *Escherichia coli* strain DH5α (Invitrogen, ThermoFisher Scientific)) was used for both cloning procedures, whereas BL21DE3Omp8 (Prilipov et al. 1998) was utilized for the expression experiments. The *Escherichia coli* strains were grown in LB medium or on LB agar plates at 37 °C with appropriate antibiotics (Sigma-Aldrich, St. Louis, MO). 100 µg/mL ampicillin, 40 µg/mL kanamycin and 25 µg/mL chloramphenicol were used for selection. The expression plasmid pET19b (~5.6 Kbp) carrying an N-terminal His-Tag® sequence followed by an enterokinase cleavage site and three cloning sites was obtained from Novagen (Madison, WI, USA). It was used in all cases as expression plasmid. In principle, the use of this plasmid allowed an easy purification of the expressed proteins by affinity chromatography. Unfortunately, this was not the case, because the His-Tag® sequence could not be cleaved. The N-terminal His-Tag® sequences were removed by the cloning procedure using primers that deleted the tag and the enterokinase cleavage site (see below).

### SDS–PAGE

Sodium dodecyl sulfate–polyacrylamide gel electrophoresis (SDS–PAGE) was performed according to the Laemmli gel system (Laemmli 1970). Proteins were separated by 8–12% SDS–PAGE under denaturing conditions (solubilized at 100 °C for 10 min in 4 × SDS sample buffer 1 before loading onto the gel) and under non-denaturing conditions (solubilized at 30 °C in 4 × SDS sample buffer without bromophenol and ß-mercaptoethanol before loading onto the gel). After electrophoresis, the gels were stained either with Coomassie or with silver (Gross et al. 1987).

### Isolation of the channel-forming activity from whole cells using detergents and purification of the channel-forming protein from *R. corynebacteroides*

The isolation of the cell wall porin was basically performed as has been described in a previous publication (Riess and Benz 2000). In brief: the cell pellet was subjected several times to a freeze–thaw cycle between −80 and 30 °C. By this method, it was possible to disintegrate the cells and to separate the cell wall from the cytoplasm by centrifugation. The channel-forming protein was extracted from the cell wall fraction using different washing steps with detergent solutions. About 2 g (wet weight) of the cell wall fraction was washed first with 10 mL 10 mM Tris–HCl, pH 8.0 supplemented with 0.2% sodium dodecyl sulfate (SDS) (buffer I) for 20 h at elevated temperature (50 °C) under agitation followed by centrifugation at 14,600×*g* for 10 min. The resulting pellet was suspended in 10 mL of a solution containing 10 mM Tris–HCl, pH 8.0 supplemented with 1% Genapol (buffer II) for 20 h at 50 °C followed by centrifugation. The channel forming activity was preferentially present in the final supernatant. SDS*–*PAGE of the proteins in the supernatant demonstrated that it contained a major protein band with an apparent molecular mass of about 130 kDa or about 20 kDa when the sample was boiled (Riess and Benz 2000). Final purification of the channel-forming protein was achieved by excision of this band from tricine-containing preparative SDS*–*PAGE and its extraction with 0.4% lauryldimethylamine oxide (LDAO), 10 mM Tris–HCl, pH 8.0. Applying the method, the protein appeared to be pure.

### Peptide sequencing

The purified protein with an apparent molecular mass of about 134 kDa was precipitated using trichloroacetic acid to remove the detergent. The amino acid sequence of the N-terminal end of the polypeptide was determined by the Edman-degradation method using a gas phase sequenator (470A, Applied Biosystems, Weiterstadt, Germany) with online detection of the amino acids.

### Cloning of the gene porARc from R. corynebacteroides and porARr and porBRr from R. ruber in the vector pET19b

The gene *porARc* without coding for the signal peptide (540 bp) was directly taken from the genome of *R. corynebacterioides* and combined with the pET19b without the nucleotides coding for the enterokinase site and the His-Tag® via Gibson assembly with 15 nucleotides of overlapping region (Gibson et al. 2009). The genome was extracted with the kit Gen-Elute (Sigma-Aldrich, St. Louis, MO) and the plasmid with kit NucleoSpin Plasmid (Artikel-Nr.: 740588.50, Macherey–Nagel, Düren, Germany), then they were both amplified via PCR in triplicate according standard PCR conditions according to the recommendations of the manufacturer (New England Biolabs). Primers to clone the genes of the cell wall channels were bought from Eurofins Genomics (Ebersberg, Germany). Primers for cloning of *porARc* are listed in Table 1. The T_*M*_ of these primers was very high therefore we performed a 2-steps PCR with annealing coupled to elongation at 72 °C for 1 min.

**Table 1:** Primers for the amplification of *porARc* and pET19b

Oligonucleotide	Sequence 5′ → 3′
pET19b-to-*porARc*-For	**CGTCGACAGCCATGG**TATATCTCCTTC (27 bp)
pET19b-to-*porARc*-Rev	**CTTCAACTGACATAT**GCTCGAGGATCC (27 bp)
*porARc*-to-pET19b-For	**CCATGGCTGTCGACG**ATTCCAACTCGGTGGTCGACGGTGGCGGC (44 bp)
*porARc*-to-pET19b-Rev	**ATATGTCAGTTGAAG**CGCCACGGCTGGCCGAACGTGGTGGC (41 bp)

The bold and underlined nucleotides represent the overlapping regions (for with for and rev with rev). The accession number of PorARc is WP_169818371.1

The gene coding for PorARr (MspA-like protein) was purchased from GeneScript (860 Centennial Ave., Piscataway, NJ 08,854, USA). It was obtained as an optimized gene in the vector pET7435 without coding for the signal peptide. The gene coding for PorBRr (porin) without signal peptide was cloned from chromosomal DNA from R. ruber DSM-43338 using the same procedure as described above for the gene coding for PorARc. Shuttle vector pET19b was used for genetic complementation and expression experiments. Besides the ampicillin resistance, the vector contained the multi cloning site (MCS) with the relevant restriction sites BamH1 and Ndel in 5′ to 3′ orientation. The genes porARr derived from the plasmid pET7435, and porBRr also without coding for the signal peptide, derived from genomic DNA of R. ruber, were amplified with standard PCR methods in 50 µL reaction volumes with 10 µL 5 × Phusion HF buffer, 0.2 mM dNTPs, 0.05 µL DMSO, 0.5 µL HF Phusion and 0.4 µM primers (see Table 2). The PCR products were digested for 20 min at 37 °C with a combination of the restriction enzymes Ndel/BamH1 and yielded the genes porARr (702 bp) and porBRr (540 bp) coding only for the mature proteins. The digestion mix of 20 µL contained 1.8 µg DNA, 0.5 µg BamHI, 0.5 µg Ndel, and 2 µL fast digest 10 × buffer. porARr and porBRr were ligated into the linearized vector pET19b with T4 DNA ligase (ThermoFisher Scientific) to yield the plasmids pET19b_porARr and pET19b_porBRr.

**Table 2:** Oligonucleotides used in this study for cloning of the *genes porARr and porBRr* in the vector pET19b

Nr	Oligonucleotide	Sequence 5′ → 3′	Proteins in* R. ruber* DSM-43338
1	FrgBNdeIR.R-F	CTT**CATATG**GCGGTCGACGATCA (23 bp)	MspA-like protein PorARr WP_003937791, 267 (234) aa
2	FrgBBamHIR.R-R	ATT**GGATCC**TCAGACGGTCCAGG (23 bp)	Porin PorBRr WP_003937792, 213 (180) aa

These genes were used for cloning of the MspA-like protein PorARr from the vector pET7435 and the porin PorBRr from the total DNA of *R. rubens* DSM-43338, respectively. Recognition sites for restriction enzymes BamHI and Ndel in pET19b are bold and underlined. The number of amino acids in the brackets refer to those of the mature proteins (see Fig. 2). The accession numbers of the proteins are WP_003937791.1 (PorARr) and WP_003937792.1 (PorBRr).

We removed the nucleotides coding for the N-terminal His-Tag® and the enterokinase site by the cloning procedure taking profit from the restriction sites BamHI and NcoI in pET19b. The corresponding primers for the mutation of pET19b in terms of *porARr* and *porBRr* are shown in Table 3. The results are pET19b_porARr* and pET19b_porBRr*.

**Table 3:** Oligonucleotides used in this study for the cloning of *porARr* and *porBRr* in the vector pET19b without the nucleotide sequence coding for the enterokinase site and the N-terminal His-Tag®

Oligonucleotide	Sequence 5′ → 3'	
Forward NcoI	CTT**CCATGG**TGGTTGATGACCAG (23 bp)	Cloning of pET19b-porARr*
Reverse BamHI	ATC**GGATCC**AAGCTTTCATTACAGG (25 bp)	
Forward NcoI	CTT**CCATGG**CGGTCGACG (18 bp)	Cloning of pET19b-porBRr*
Reverse BamHI	ATT**GGATCC**TCAGACGGTCCAGG (23 bp)	

Recognition sites for restriction enzymes BamHI and NcoI are bold and underlined

### Expression of PorARc, PorARr, and PorBRr in the porin deficient BL21DE3Omp8 *E. coli* strain

Several protein subunits of cell wall channels of members of the mycolata were previously successfully expressed in porin-deficient *E. coli* species (Niederweis et al. 1999; Kläckta et al. 2011). After a renaturation procedure, these subunits formed oligomers. Examples are the MspA monomer of *M. smegmatis* and the NfpA and NfpB monomers of *N. farcinica* (Niederweis et al. 1999; Kläckta et al. 2011; Singh et al. 2015) that formed channels in lipid bilayer membranes. Genes *porARc*, *porARr* and *porBRr* were cloned by inserting in pET19b, resulting in the vectors pET19b_*porARC*, pET19b_porARr*, and pET19b_porBRr*. The ligation products were transformed into porin deficient *E.coli* BL21DE3Omp8 cells (Prilipov et al. 1998) via a slightly modified standard electro-transformation method via electroporation at 600 Ω, 25 µF and 2.5 kV in multiporator (Eppendorf)*.* All plasmids were checked by sequencing prior to transfection of BL21DE3Omp8 *E. coli* cells. Similarly, the correct orientation of the genes was also confirmed by sequencing (GATC Biotech Sequencing Center, Köln, Germany).

### Isolation and purification of PorARc, PorARr, and PorBRr

The porin deficient BL21DE3Omp8 *E. coli* cells containing the expression plasmids pET19b_*porARc*, pET19b_porARr*, or PET19b_porBRr* were grown at 37 °C in LB medium to an OD_600_ of about 0.6 using the appropriate selection of antibiotics. Then expression was induced at room temperature by adding 1 mM isopropyl β-d-1-thiogalactopyranoside (IPTG, Sigma-Aldrich, St. Louis, MO) to the culture media. After 16 h, cells were collected by centrifugation at 10,000×*g* for 10 min at 4 °C and the pellet was stored at −20 °C for the extraction steps. The frozen *E. coli* BL21DE3Omp8 cells were thawed and resuspended in 10 mL 10 mM Tris–HCl pH 8.0 containing 10 μg/mL of pancreatic DNase I and 100 μg/mL of RNase A (Sigma-Aldrich, St. Louis, MO) and 1 mM phenyl methyl sulphonyl fluoride (PMSF – Carl Roth, Karlsruhe Germany). Protease inhibitor cocktail (Merck, Darmstadt, Germany) was added before disrupting the cells 5-times by a French pressure cell on ice. Cell debris was collected by centrifugation at 5,000×*g* for 30 min at 4 °C. The supernatant was ultra-centrifuged at 100,000×*g* for 1 h at 4 °C to obtain the membrane pellet. The supernatant (US1) contained the soluble part of the three proteins. The membrane pellet was resuspended in 10 mM Tris, 3% octyl-POE (Sigma-Aldrich, St. Louis, MO) and protease inhibitor cocktail set II (Merck, Darmstadt, Germany) was added before shaking the suspension for 30 min at RT followed by ultra-centrifugation at 100,000×*g* for 1 h at 4 °C yielding the supernatant US2. Supernatants and pellets were separated and stored for analysis with 10% SDS-PAGE (Laemmli 1970). The supernatant contained the three mature proteins PorARc, PorARr, and PorBRr.

Protein samples that showed the desired protein bands on SDS-PAGE were collected and concentrated (Amicon® Ultra 15 mL Centrifugal Filter’s cutoff 50 KDa (Sigma-Aldrich, St. Louis, MO)). The proteins were then loaded on a FPLC (Fast-Protein Liquid chromatography) (Biorad, Germany) Source 15Q anion exchange column (GE Healthcare, München, Germany) equilibrated with a buffer containing 20 mM Tris pH 8, 0.5% (v/v) octyl-POE. The proteins were eluted gradually with increasing NaCl concentration from 0 to 500 mM in fractions of 1 mL for 10 column volumes. All three proteins (PorARc, PorARr and PorBRr) eluted around 450 mM NaCl in a very sharp peak. SDS-PAGE revealed that the proteins were essentially free of contaminant proteins.

### Mass spectrometric identification of proteins from SDS-PAGE bands

The Coomassie-stained 1D-SDS gel bands which contained either PorARc, PorARr, or PorBRr were independently worked up to generate tryptic peptide mixtures according to published protocols (Sinz et al. 2002; Konus et al. 2013; Röwer et al. 2018). Mass spectrometric analysis of peptide mixtures was performed on a Synapt G2-S mass spectrometer (Waters, Manchester, UK) coupled to a nanoAcquity UPLC system (Waters MS-Technologies, Manchester, UK) via a NanoLockSpray ion source using a PicoTip Emitter (New Objective, Woburn, MA, USA) as described elsewhere (Röwer et al. 2018; Kumar et al. 2018b).

For automated protein identification, MS^E^ data were processed using ProteinLynx GlobalSERVER version 2.3 (Waters MS-Technologies). Protein identifications and partial amino acid sequence assignments were obtained by searching against all entries of a manually curated database with sequence entries from *Rhodococcus corynebacteroides and Rhodococcus ruber* which were extracted from the UniProt/Swiss-Prot (UniProt release 2021_01) database to which the sequence information of trypsin (*Sus scrofa*) plus all reviewed proteins from *Homo sapiens sapiens* were manually added (Röwer et al. 2009; Postu et al. 2019). Search parameters were set to: four missed cleavage sites, oxidation of methionine residues as variable modification, and carbamidomethylation of cysteines as fixed modification. Peptides were identified by at least three fragment ions. Singly charged peptide ions were rejected, whereas peptides with two, three, and four positive charges were accepted. Furthermore, peptides were removed from the hit list that had (i) a peptide score below 5.5, (ii) a mass error above 13 ppm, and (iii) less than six amino acid residues in length.

For manual protein identification, MS^E^ data were processed using MassLynx version 4.1 (Waters MS-Technologies). Protein identifications and partial amino acid sequence assignments were obtained by calculating the masses of the predicted tryptic peptides and their fragment ion signals from the provided amino acid sequence information (Röwer et al. 2009; Mádi et al. 2008). Amino acid sequence coverages were graphically displayed on the PorARc, PorARr, or PorBRr protein sequences.

### Renaturation of PorARc, PorARr, and PorBRr

Proteins from the cell wall of members of the taxon mycolata need very often some sort of renaturation before they could successfully be used for the study of channel-formation in lipid bilayers (Niederweis et al. 1999; Kläckta et al. 2011). This seemed not to be necessary here because when the purified proteins were simply dissolved in detergent solution, PorARc, PorARr and PorBRr alone and a combination of PorARr and PorBRr, formed channels in polarized lipid bilayer membranes.

### Planar lipid bilayer assay

The methods used for black lipid bilayer experiments have been described previously in detail (Benz et al. 1978, 1979). The instrumentation consisted of a Teflon chamber with two water-filled compartments separated by a thin wall and connected by a small circular hole with an area of 0.4 mm^2^. The membranes were formed from a 1% (w/v) solution of diphytanoyl phosphatidylcholine (DiPh-PC) (Avanti Polar Lipids, Alabaster, AL) in *n*-decane by painting onto the hole a 1% (w/v) solution of the lipid in n-decane. The protein-containing fractions were added to the aqueous phase after the membrane had turned black to one side of the membrane (the cis-side). The membrane current was measured with a pair of Ag/AgCl electrodes with salt bridges switched in series with a voltage source and a highly sensitive current amplifier (Keithley 427, Cleveland, Ohio). The temperature was kept at 20 °C throughout. Selectivity measurements were performed by establishing a fivefold KCl-gradient (0.1 M versus 0.5 M KCl) across lipid bilayer membranes containing about 100–1000 pores formed either by PorARc, PorARr, and PorBRr alone or by a combination of PorARr and PorBRr. The zero-current membrane potentials were measured with a Keithley 617 electrometer and analyzed using the Goldman–Hodgkin–Katz equation (Benz et al. 1979).

## Results

### Identification of genes coding for elements of the cell wall channel in the genome of R. ruber

**Fig. 1:**
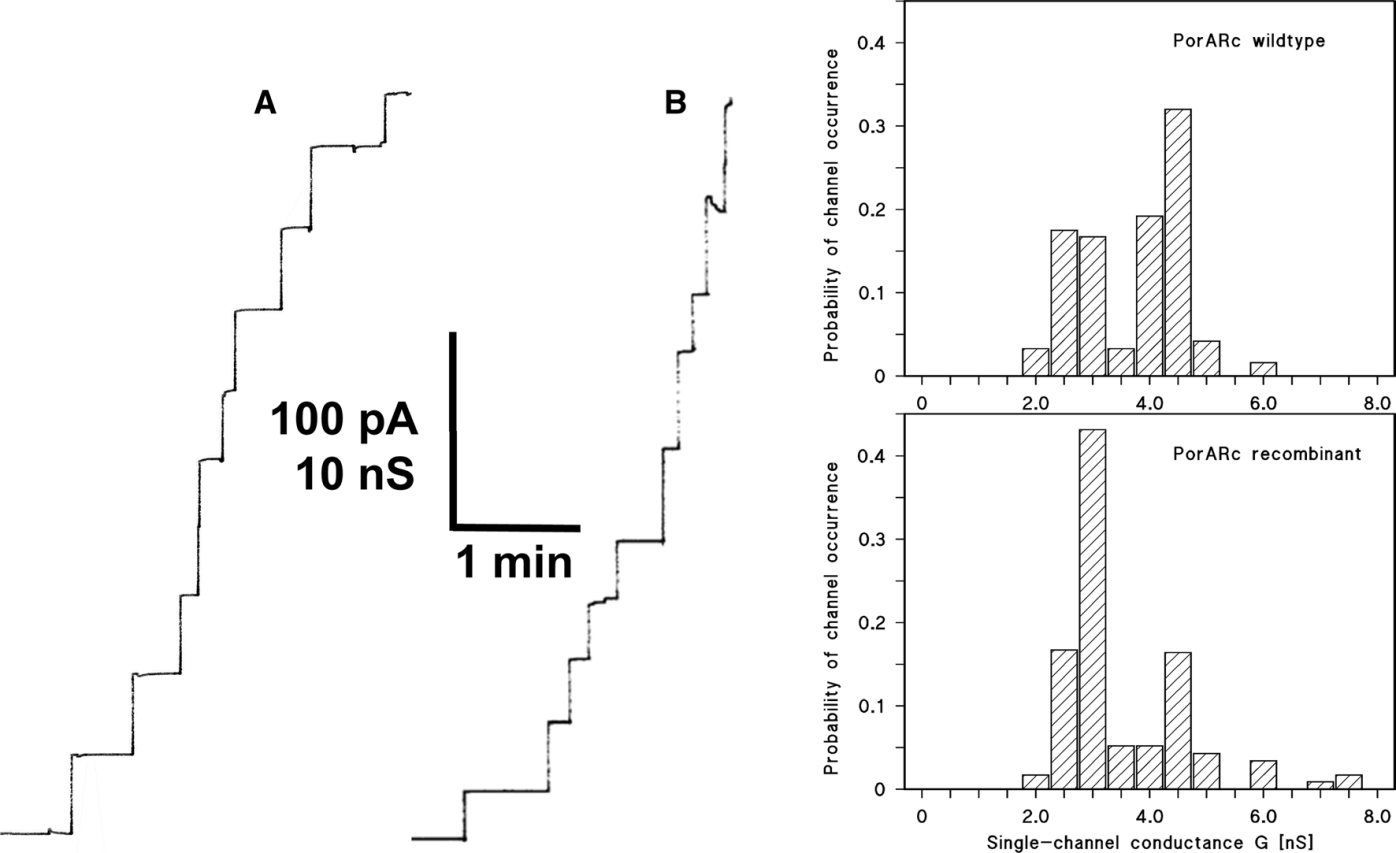
Current recordings of wildtype PorARc (**A**) and recombinant PorARc (**B**) of *R. corynebacteroides* reconstituted in DiPh-PC/*n*-decane membranes. **A** The aqueous phase contained 1 M KCl, pH 6.0, as electrolyte and about 0.7 nM wt. PorARc isolated from *R. corynebacteroides*. The average single-channel conductance was 3700 ± 470 pS for 120 single events taken from five individual membranes. **B** The aqueous phase contained in addition to the electrolyte about 10 nM recombinant PorARc. The average single-channel conductance was 3560 ± 510 pS for 116 single events taken from seven individual membranes. The applied membrane potential was 10 mV; *T* = 20 °C

In a previous study a channel-forming protein was identified in the cell wall of *Nocardia corynebacteroides* NCTC 10391 (Riess and Benz 2000), later on reclassified as *Rhodococcus corynebacteroides* ATCC 14898 (Yassin and Schaal 2005). This protein, termed here PorARc (for **PorA** of ***R****. ****c****orynebacteroides*) had an apparent molecular mass of 134 kDa on SDS-PAGE when it was solubilized at 40 °C. The 134 kDa protein PorARc dissociated into 23 kDa subunits when it was heated to 100 °C for 10 min in sample buffer (Riess and Benz 2000). We repeated isolation and purification of the 134 kDa oligomeric protein from *R. corynebacteroides* DSM-20151 using essentially the same procedure as published previously (Riess and Benz 2000). The purified protein oligomers were active in the lipid bilayer assay and formed pores with a single-channel conductance of 3.0 and 4.5 nS in 1 M KCl, pH 6.0 solution (see Fig. 1). For the selectivity measurements, we established a fivefold KCl-gradient across lipid bilayer membranes containing 50–100 PorARc pores. The asymmetry potential under these conditions was 22 ± 2.5 mV (mean of three experiments). Analysis of the potential using the Goldman–Hodgkin–Katz equation (Benz et al. 1979) suggested that the ratio of the permeability *P*_cation_ and *P*_anion_ was about 4.0 for KCl, which is in good agreement with the previous data (*P*_K_/*P*_Cl_ = 3.8; Riess and Benz 2000). These results indicated that we isolated in this study the same cell wall channel as previously.

The oligomeric 134 kDa protein was partially sequenced by N-terminal Edman-degradation performed by Hartmut D. Kratzin from the Max-Planck-Institute for Theoretical Medicine, Göttingen, Germany. A sequence of 20 amino acids of the N-terminus of the prospective polypeptide subunit was obtained by this procedure: AVDDSNSVVDGGGNTITVSQ, without any indication of uncertainties with respect to amino acids. This means that the N-terminal end of PorARc from the *R. corynebacteroides* DSM-20151 was identified by the partial polypeptide sequence without indication for a second sequence. We used the partial sequence for a BLAST search of the corresponding proteins (https://blast.ncbi.nlm.nih.gov/Blast.cgi), (Altschul et al. 1990, 1997). It suggested that the subunit of the cell wall channel of *R. corynebacteroides* is a member of the superfamily of MspA-like cell wall proteins (MspA family porin; WP_169818371.1). These proteins form channels responsible for the cell wall permeability of a large number of Gram-positive bacteria of the mycolata taxon, also known as *Nocardia*-*Mycobacterium*-*Corynebacterium* (NMC) complex (Niederweis et al. 1999; Stahl et al. 2001; Dörner et al. 2004; Kläckta et al. 2011). Interestingly, the partial sequence of PorARc was homologous to many hypothetical proteins of *Rhodococcus* species (more than 58 hits in Protein-BLAST). The pore subunits of Rhodococcus belong to the MspA family of proteins designated as PF09203 in Pfam; (https://pfam.xfam.org/family/PF09203).

**Fig. 2:**
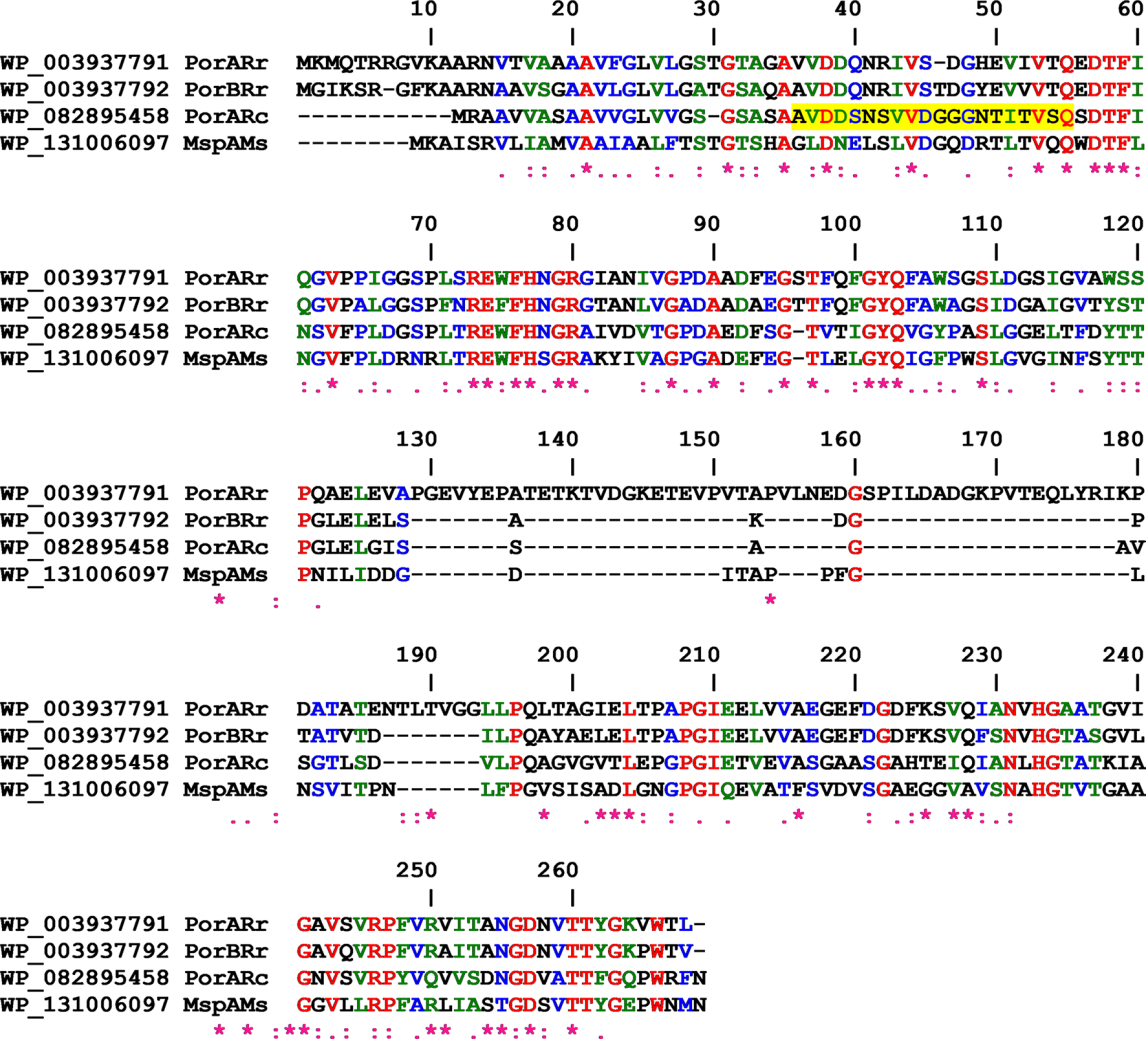
Amino acid sequence alignment of PorARr, PorBRr of *R. ruber*, PorARc of *R. corynebacteroides* and MspA of *M. smegmatis. *The alignment was performed using the indicated NCBI Reference protein sequences and Pole Bioinformatique Lyonnaise Network Protein Sequence Analysis (http://npsa-pbil.ibcp.fr). Amino acids identical in all three proteins are highlighted in red (*), strongly similar amino acids (:) are given in green and weakly similar ones (.) in blue. The yellow highlighted sequence was found by Edman-degradation of the N-terminal end of PorARc of *R. corynebacteroides*. It is noteworthy that all these ORF code for proteins with a signal peptide indicating that the proteins are exported out of the cell using the *Sec* apparatus. The cleavage site for the signal peptide of PorARc is clear from its N-terminal sequencing suggesting that all the mature proteins start with the amino acid in position 36 in this figure

**Fig. 3:**
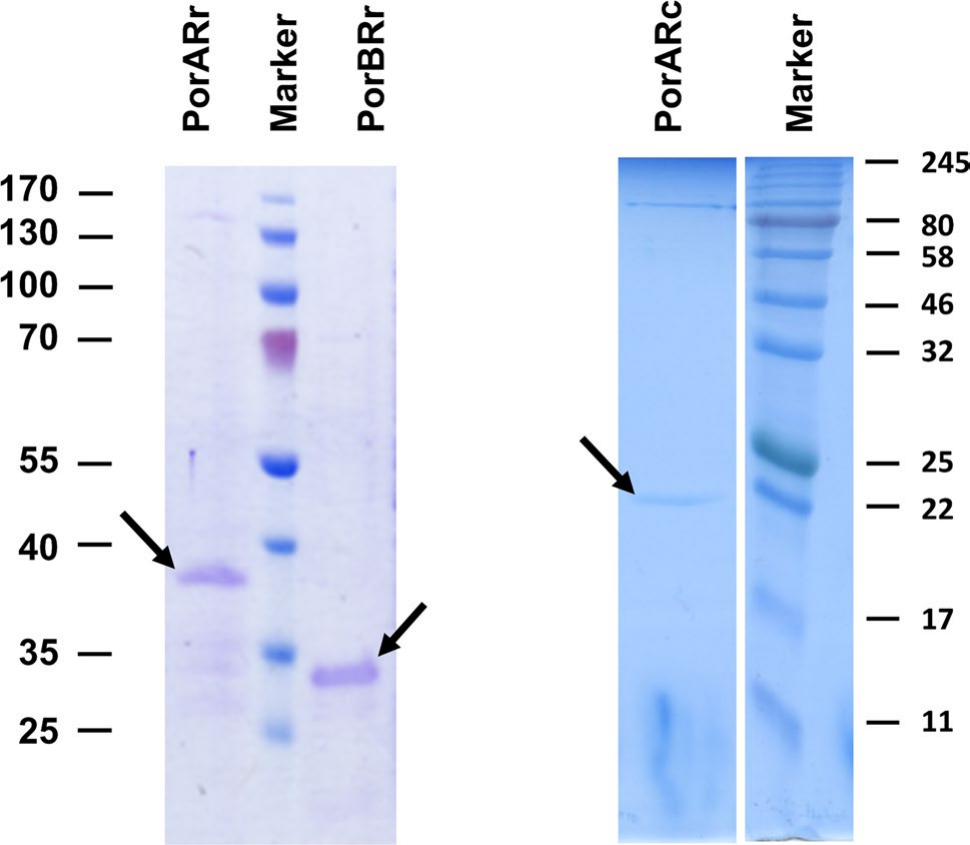
12% SDS-Page of the two-polypeptide subunits of the cell wall channel of *R. ruber* (left side arrows) and the subunit of *R. corynebacteroides* (right side arrow). Left side; subunits of *R. ruber*: The central lane shows molecular mass markers: 170, 130, 100, 70, 55, 40, 35, and 25 kDa. The left lane shows PorARr purified according to the method described in experimental conditions with an apparent molecular mass around 38 kDa. The right lane shows PorBRr purified as described with an apparent molecular mass of about 30 kDa. Right side; subunit of *R. corynebacteroides* cell wall channel: The right lane shows molecular mass markers: 245, 190, 135, 100, 80, 58, 46, 32, 25, 22, 17 and 11 kDa. The protein samples were heated to 100 °C in sample buffer before being loaded on the gels. The gels were stained with Coomassie brilliant blue

The cell wall channel of *R. corynebacteroides* has in particular a high homology to hypothetical proteins of *R. fascians* (genome ID: 11595) and *R. ruber* BKS 20–38 (genome ID: 11562) (Bala et al. 2013). The genome of *R. ruber* contained two open reading frames that were able to code for channel-forming proteins, MspA-like protein WP_003937791 (267aa) and porin WP_003937792 (213aa) (see the alignment of the different MspA-like proteins in Fig. 2). The genetic organization of the genes coding for the two ORF is such that the two genes *porARr* (for ***porA*** of ***R****. ****r****uber*) and *porBRr* (for ***porB*** of ***R****. ****r****uber*) are organized in pairs arranged directly behind one another. They are presumably transcribed together, which means that both proteins could be needed to form an active oligomeric cell wall channel. Both proteins contain also a signal peptide, which means that they are transported via the Sec-system out of the cell. Figure 2 shows an alignment of the MspA porin of *R. ruber* (named **PorARr**) with the porin (named **PorBRr**), the cell wall protein of *R. corynebacteroides* (**PorARc**) and MspA of *M. smegmatis* (**MspAMs**). The interesting point of this alignment is that the porin PorBRr, MspA and PorARc of *R. corynebacteroides* have approximately the same length, whereas PorARr of *R. ruber* is much longer because of an insertion of about 60 amino acids between aa 130 and aa 190. Subsequently, we expressed the genes of PorARc and the two cell wall proteins from *R. ruber* (PorARr and PorBRr) in a porin-deficient *E. coli* strain (BL21DE3Omp8), purified the proteins to homogeneity and studied their pore-forming properties in the lipid bilayer assay. Figure 3 shows SDS-PAGEs of both purified putative subunits of the *R. ruber* cell wall channel and PorARc. The purity of the three proteins was also checked by mass spectrometry following digestion of the protein bands with trypsin or elastase. The score and number of significant matches was in all three cases much higher for the porins than for the proteins with the next score or significant matches, either trypsin or elastase (see Supplemental Table S1 of Supporting Information).

### Mass spectrometric identification of “cell wall proteins” from SDS-PAGE bands

As in trans-membrane proteins, such as channel proteins, numbers of cleavage sites for trypsin were found to be too scarce in order to generate peptide mixtures with enough peptides and such with suitable sizes for sufficient liquid chromatography separation and with optimal peptide ion masses, mass spectrometric identification of cell wall proteins with standard “peptide mass fingerprint” protocols was not successful. Instead, for precise identification of channel proteins after in-gel digestions, a specifically adapted three-step protocol was required to generate convincing identification results (see “Supporting Information; Mass Spectrometry Supplement”). Thus, in this project the targeted “peptide mapping” strategy was mandatory for cell wall protein identification.

### Pore formation in lipid bilayer membranes by recombinant PorARc

PorARc was recombinantly expressed in *E. coli* BL21DE3Omp8, isolated and purified as described. Addition of small amounts of the protein added to black lipid bilayer membranes made of DiPh-PC/*n*-decane membranes resulted in a rapid reconstitution of membrane pores as shown in Fig. 1. Conductance and characteristics of the pores formed by recombinant PorARc were similar to those of the wildtype protein, as shown in Fig. 1. Only the histograms of the two types of pores showed some differences (see Fig. 1). Protein isolated from *R. corynebacteroides* formed pores with a preference for pores with a conductance of 4 to 5 nS in 1 M KCl. Pores formed by the recombinant protein had a preference for a conductance of 2.5 to 3.5 nS, although both conductance values were present in the experiments (see histograms in Fig. 1).

### Pore formation in lipid bilayer membranes was possible by PorARr alone and by PorARr of *R. ruber* alone and by combinations of PorARr and PorBRr

**Fig. 4:**
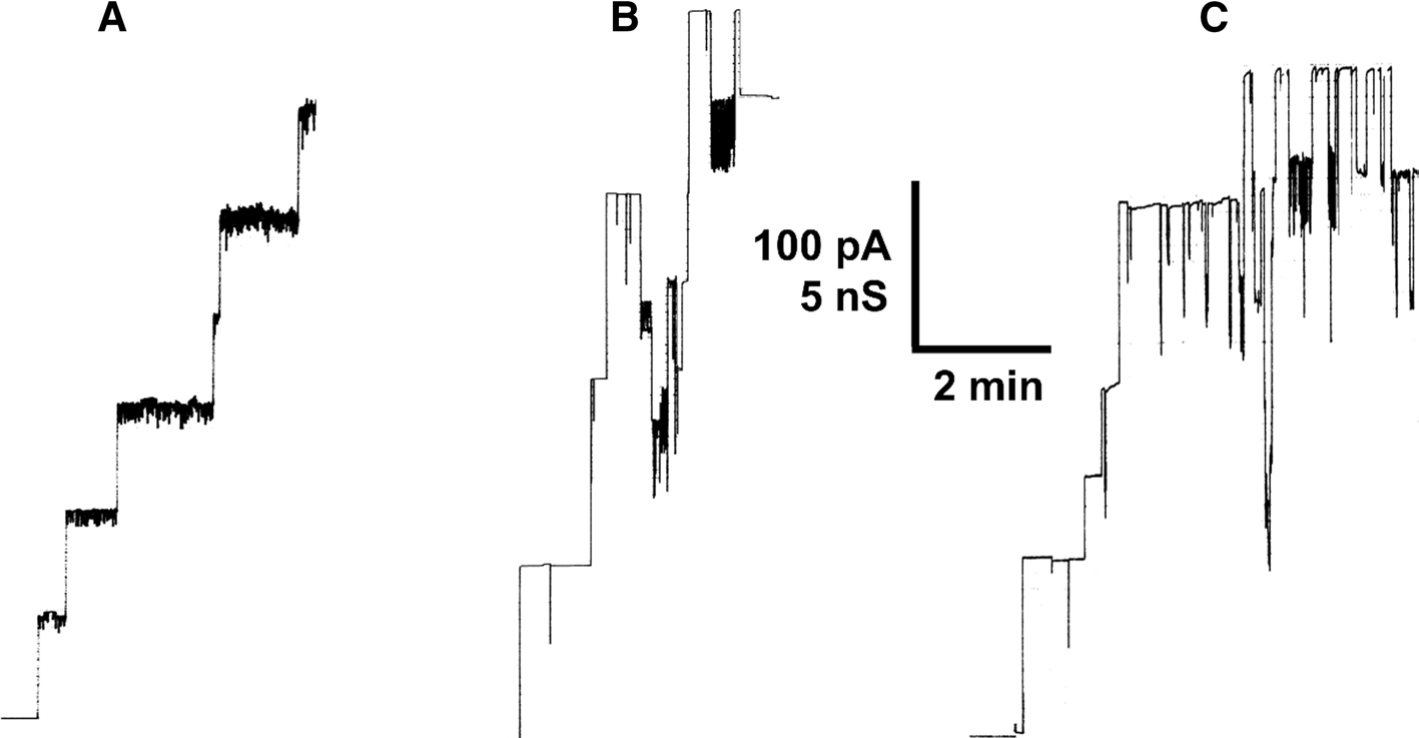
Current recordings of DiPh-PC/*n*-decane membranes after addition of purified recombinant PorARr (**A**), PorBRr (**B**) and a combination of PorARr and PorBRr in a molar ratio of 1:1 (**C**) to the cis-side of different DiPh-PC/*n*-decane membranes. The aqueous phase contained 1 M KCl, (pH 6) and about 1 nM of the proteins immersed in detergent solution added to cis-sides of the black membranes. The applied membrane potential was 20 mV; *T* = 20 °C

**Fig. 5:**
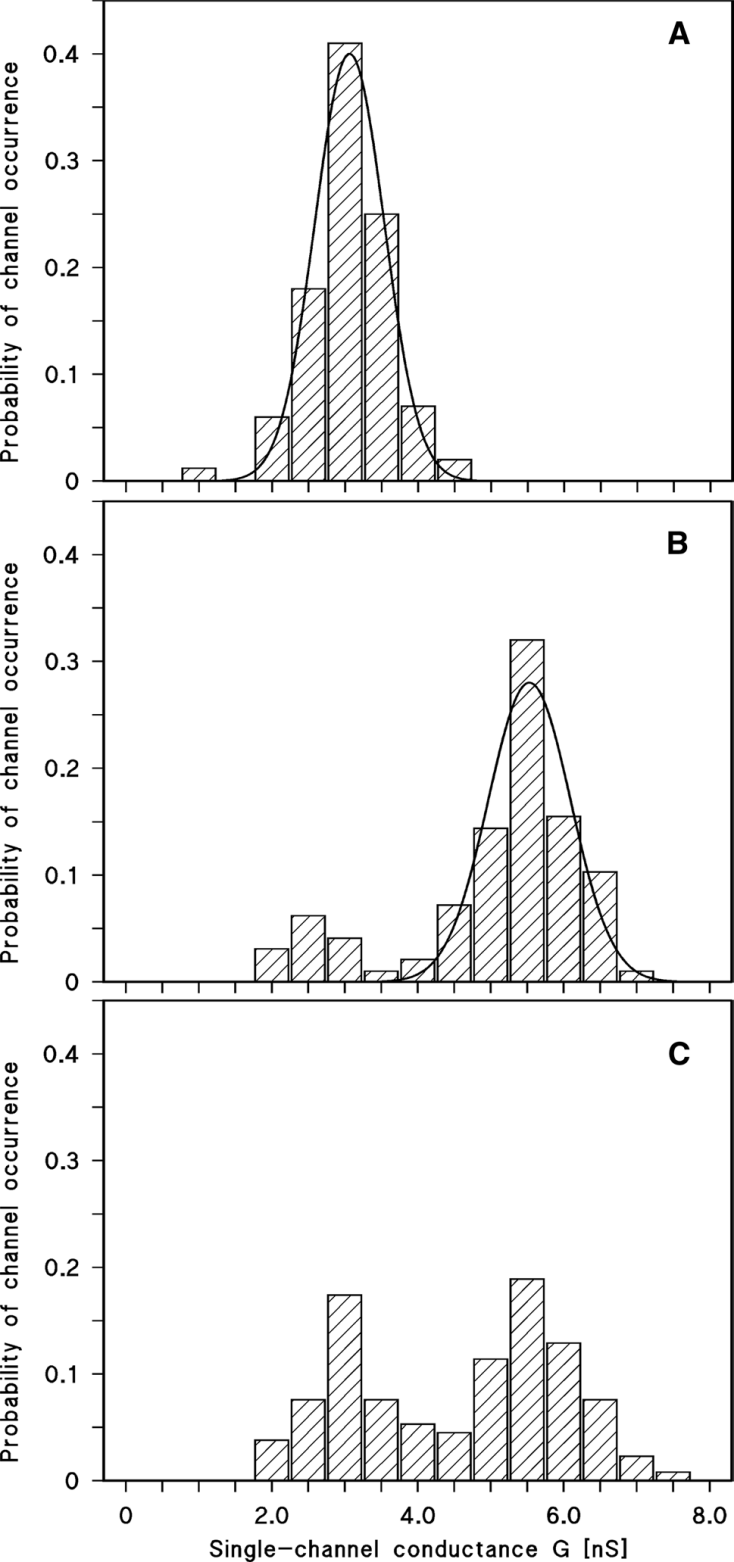
Histograms of the probability of pore-formation by PorARr, PorBRr, and a 1:1 mixture of both polypeptides in DiPh-PC/*n*-decane membranes and 1 M KCl, pH6 as electrolyte. **A** Measurements with PorARr; the solid line shows a fit of the histogram with a Gaussian distribution. The maximum of the distribution is at a probability of 0.40 ± 0.015 and the conductance is 3060 ± 470 pS for 128 single events taken from six individual membranes. *V*_m_ = 20 mV; *T* = 20 °C. **B** Measurements with PorBRr; the solid line shows a fit of the histogram with a Gaussian distribution. The maximum of the distribution is at a probability of 0.28 ± 0.03 and the conductance is 5530 ± 560 pS for 134 single events taken from eight individual membranes. *V*_m_ = 20 mV; *T* = 20 °C. **C** Measurements with PorARr mixed with PorBRr in a relation 1:1. The histogram shows two maxima indicating a heterogeneous distribution of channels, which did not allow to be fitted to a Gaussian distribution of all pores. The average single channel conductance of 186 pores was 4610 ± 680 pS taken from seven individual membranes. *V*_m_ = 20 mV; *T* = 20 °C

The genes coding for PorARr and PorBRr are organized in tandem and are presumably transcribed together. Therefore, the question arises whether the cell wall channels of *R. ruber* are composed of both subunits or from only one alone, either PorARr or PorBRr. To answer this question, both subunits were separately expressed in *E. coli* BL21DE3Omp8 without the N-terminal His-Tag®. Proteins were purified by chromatography across a Source 15Q anion exchange column to homogeneity (see Fig. 3). Interestingly, both single polypeptides PorARr alone and PorBRr alone dissolved in detergent solution were able to form pores in the lipid bilayer assay (see Fig. 4A, B). This was surprising because other polypeptides forming cell wall pores of bacteria from the order *Corynebacteriales* (*i.e.,* the NMC-complex) form only channels when both subunits were present as has been demonstrated for PorA and PorH of *Corynebacterium glutamicum* and NfpA and NfpB of *Nocardia farcinica* (Barth et al. 2010; Kläckta et al. 2011). The pores formed by PorARr were found to be quite homogeneous, as the single-channel recording and the histogram of Fig. 5A clearly indicates. Only the open pores showed some flickering, which increased with their number.

Channel formation was also observed when PorBRr was added to the cis-sides of black DiPh-PC/*n*-decane membranes. The pores showed in this case a somewhat different appearance as in the case of PorARr (see Fig. 4B). The single-channel conductance of the pores formed by PorBRr alone was for most of the conductance fluctuations between 5.0 and 6.0 nS (about 70% of the events), whereas only 12% of the events had a conductance of about 3.0 nS in 1 M KCl (see Fig. 5B). Interestingly, the channel switched very often in substates that could be responsible for the maximum around 3 nS in the histogram (see Figs. 4B and 5B). Current steps with a single-channel conductance of 5 nS in 1 M KCl were **c**haracteristic for pore formation by polypeptides of the MspA-family of proteins from Gram-positive bacteria of the NMC-complex (Trias et al. 1992; Trias and Benz 1993; Riess et al. 1998; Kläckta et al. 2011). Similarly, these pores switched also in substates with a conductance of about 3 nS as found for PorBRr (see Fig. 5B).

Channel forming activity was also observed when both recombinant *R. ruber* proteins (PorARr and PorBRr) were mixed 1:1 in detergent solution and added together to the membranes (see Fig. 4C). The histogram of these current steps showed a high heterogeneity with conductance maxima around 3 and 5 nS (see Fig. 5C). This was unsurprising because addition of pure PorARr and PorBRr polypeptides resulted in similar conductance steps (see Fig. 4). Treatment of the PorARr and PorBRr mixtures similarly as previously performed with MspA of *M. smegmatis* or the two subunits of *N. farcinica*, i.e. precipitation with saturated ammonium sulfate followed by detergent treatment, did not influence the histogram. Thus, it seems very likely, that even the mixed protein sample shows two pore types: one that consisted only PorARr and another that consisted only PorBRr, i.e. by separate pore insertions of PorARr and PorBRr. This is presumably also connected to the mechanism of pore formation in the mycolic acid layer of the NMC-complex, which is largely unknown, but occurs definitely differently than in lipid bilayer membranes (see Fig. 5). Other ratios of PorARr with PorBRr resulted also in similar pore formation with some change of the magnitude of the maxima. However, the broad distribution of pores over the range of about 2.5–6 nS was found for all these mixtures.

### Analysis of the pores formed by PorARr and PorBRr alone and a combination of the two polypeptides

Pore-formation of PorARr and PorBRr and mixtures of both polypeptides were also studied in different electrolytes and in different KCl-concentrations. The results are summarized in Table 4. The single-channel conductance of the pores formed by the two polypeptides and their mixture indicated that all pores had a preference for cationic solutes, because conductance in KCl and KCH_3_COO was very similar, whereas it was much lower in 1 M KCl. This is typical for most of the cell wall channels of bacteria from the NMC-complex (Trias and Benz 1993, 1994; Riess et al. 1999; Niederweis et al. 1999; Kläckta et al. 2011). The dependence of the single-channel conductance was not a linear function of the KCl-concentration for all three systems (PorARr, PorBRr and PorARr mixed with PorBRr). This has also been found for cell wall channels of bacteria of the NMC-complex. Ion transport through the channels is controlled by hot spots of negatively charged groups near the periplasmic pore entrance (Trias and Benz 1993, 1994; Riess et al. 1998; Niederweis et al. 1999; Faller et al. 2004; Kläckta et al. 2011).

**Table 4:** Average single-channel conductance of pores formed by PorARr, PorBRr, and a 1:1 mixture of PorARr and PorBRr of *R. ruber* in different electrolyte solutions

Electrolyte	Concentration (M)	PorARr	PorBRr	PorARr and PorBRr
		G (pS)		
KCl LiCl KCH_3_COO (pH 7)	0.03 0.10 0.30 1.00 3.00 1.00 1.00	320 ± 70 620 ± 95 1023 ± 110 3040 ± 230 6400 ± 255 1060 ± 180 2960 ± 210	450 ± 60 900 ± 110 1850 ± 120 5530 ± 570 11,200 ± 650 1950 ± 120 4850 ± 430	400 ± 90 750 ± 120 1280 ± 320 4610 ± 680 8500 ± 1200 1500 ± 200 4100 ± 710

The membranes were formed from DiPh-PC/*n*-decane. The single-channel conductance was measured at 20 mV applied voltage and *T* = 20 °C. About 1 nM of PorARr, PorBRr and a 1:1 mixture of the two polypeptides were added to the cis-side of the membranes. The average single-channel conductance, G (±SD), was calculated from at least 100 single events, either by fitting the histograms by Gaussian distributions (similar to Fig. 5A, B) or by averaging over all single conductance steps (Fig. 4C).

### Ionic selectivity of the pores formed by PorARr and PorBRr

We performed zero-current membrane potentials to confirm the cation selectivity of the pores formed by PorARr and PorBRr alone and by mixtures of both proteins. After the incorporation of 100 to 1000 pores into the DiPh-PC/*n*-decane membranes, the salt concentration on one side of the membranes was raised fivefold from 100 to 500 mM by adding small aliquots of 3 M KCl solution to one side of the membranes. Zero-current potentials were measured 5 min after the gradients were established. The more dilute side (100 mM) always showed positive potential, irrespective of whether the pores were formed by PorARr, PorBRr or by 1:1 mixtures of both proteins. This indicated preferential movement of potassium ions through the pores. The zero-current membrane potentials for fivefold gradients of KCl were on average (3 measurements) around 33 ± 2 mV (PorARr), 31 ± 3 mV (PorBRr) and 32 ± 2 mV (PorARr and PorBRr in 1:1 relation) on the more diluted side of the membrane. Analysis of these data using the Goldman-Hodgkin-Katz equation (Benz et al. 1979) suggested that the selectivity of the pores was very high and reached values for the ratios of the permeability *P*_K_ and *P*_Cl_ around 10 and higher indicating that the pores had little or almost no permeability for chloride, which is typical for pores formed by cell wall channels of the NMC-complex (Trias and Benz 1994; Lichtinger et al. 2000 ; Riess et al.^1998^; Niederweis et al. 1999; Riess and Benz 2000; Kläckta et al. 2011; Mafakheri et al. 2014). On the other hand, it is possible that the cell walls of the NMC-complex contain in addition to the cation-selective channels also anion-selective ones because this has been demonstrated for *R. equi* and *C. glutamicum* (Riess et al. 2003; Costa-Riu et al. 2003).

## Discussion

### The cell wall channel of *R. *corynebacteroides is formed by a single polypeptide

In a previous study, we investigated the pore-forming properties of the cell wall channel of *Nocardia corynebacteroides*, which was renamed later to *Rhodococcus corynebacteroides* (Riess and Benz 2000; Serrano et al. 1972; Yassin and Schaal 2005). Isolation and purification of the protein was repeated in this study and pore-formation was checked in lipid bilayer membranes. Pore-forming characteristics of purified PorARc in lipid bilayer membranes was very similar to the previously obtained results (Riess and Benz 2000), with the exception that the maximum of the distribution shifted from about 4.5 nS for the native cell wall channel to about 3 nS for the recombinantly expressed one. This means that the protein isolated from *R. corynebacteroides* has a prevalence to reconstitute in the larger version of the pore. The difference between both versions is not clear but it may be caused by some variations in the pore structure, which may have to do with the yet unknown translation of the polypeptides and the pore assembly in vivo. It may also be caused by post-translational modification of the pore-forming polypeptides, as has been shown for PorA and PorH of *C. glutamicum* by O-mycoloylation (Huc et al. 2010).

The N-terminal end of the pore-forming protein was subjected to Edman-degradation and amino acid analysis. Twenty amino acids were identified without any indication of ambiguities. A BLAST search clearly revealed PorARc as a member of the MspA family of subunits of cell wall channels, originally observed in *M. smegmatis* (Altschul et al. 1990, 1997; Niederweis et al. 1999). A gene coding for a second MspA-like protein was not found in the genome of *R. corynebacteroides*, which indicates that its cell wall channel is formed by a single polypeptide subunit. MspA-like proteins are widely distributed among subunits of the cell wall channel of bacteria from the NMC-complex, but also *Tsukamurella* and *Dietzia* species contain cell wall channels formed by polypeptides from the same superfamily of proteins (Dörner et al. 2004; Mafakheri et al. 2014). Here, we could identify besides PorARc also proteins of other *Rhodococci* (*R. ruber* and *R. fascians*) as members of the same superfamily of proteins, which was designated as PF09203 in Pfam.

### The genome of *R. ruber* contains the genes coding for two homologous proteins

BLAST search with the partial sequence of *R. corynebacteroides* revealed high homology with two proteins of *R. ruber* DSM-43338 (Altschul et al. 1990, 1997). One of them (WP_003937791) is designated as MspA-like protein with a length (including signal peptide) of 267 amino acids. The other one (WP_003937792) was previously designated as porin (213 amino acids including signal peptide) and now as MspA family porin in NBCI data bank. The genes coding for both proteins are organized in pairs that are presumably transcribed together, because one gene is located next to the 5′ ribosome binding site and the two genes *porARr* and *porBRr* are located directly behind one another and may form presumably some kind of operon. This arrangement suggests that both proteins are needed to form an active cell wall channel, as has been found for different strains of *Corynebacteriae* and *Nocardiae* (Barth et al. 2010; Kläckta et al. 2011). To see if a similar requirement is needed for the two porin subunits of *R. ruber*, we expressed them without signal peptide in a porin-deficient *E. coli* strain (BL21DE3Omp8).

### Pores were formed by PorARr alone and PorBRr alone

We performed lipid bilayer experiments with both proteins and demonstrated that they created pores with properties that are very similar to those of other cell wall channels of the NMC-complex (Trias and Benz 1993, 1994; Riess et al. 1998, 2001; Niederweis et al. 1999; Dörner et al. 2004). The interesting results of these experiments was that the two proteins formed homooligomeric pores. This suggested that the organization of the cell wall channel proteins in *R. ruber* is similar to that in *M. smegmatis*, where four different homologous genes (*mspA*, *mspB*, *mspC,* and *mspD*) code presumably for homooligomeric or heterooligomeric cell wall channels (Stahl et al. 2001). The single-channel conductance of the homooligomeric pores from PorARr and PorBRr differed somewhat from one another. In particular, PorBRr formed pores that switched very often into substates even at low voltages, which we did not observe for PorARr. The mixture of both proteins formed pores that showed a very broad distribution of pores, looking like a combination of the distributions of the single proteins. Attempts to change the distribution by recipes applied previously to heterologously-produced subunits of cell wall channels did not change the distribution much. This included ammonium sulfate precipitation of the 1:1 mixed proteins followed by solution in detergent (Faller et al. 2004; Kläckta et al. 2011).

The pores formed by PorARr and PorBRr of *R. ruber* in lipid bilayer membranes were cation selective. This is typical for cell wall channels from bacteria of the NMC-complex (Trias and Benz 1993; Lichtinger et al. 1998; Riess and Benz 2000), but also anion-selective channels may be present in the cell walls of *Corynebacteria* and *Rhodococci* (Costa-Riu et al. 2003; Riess et al. 2003). The 3D-structure of MspA has a homooctameric goblet-like conformation with a single central channel. The narrow opening of the channel towards the periplasmic space is lined up with 16 aspartates (eight times D90 and D91 of the single mature protein) (Faller et al. 2004). Because of the similarity of the primary sequence between MspA and the two subunits of the cell wall channel of *R. ruber* it is possible that their 3D-structures are quite similar (see Fig. 2). PorARr and PorBRr have in a similar position one glutamic acid (E90 and E91, respectively of the mature protein) with another one nearby (E88 and E89, respectively). These many negatively charged glutamates are presumably also localized near the periplasmic opening of the pores formed by oligomers of PorARr and PorBRr. They could be the reason for the high cation selectivity of the pores formed by PorARr and PorBRr and by mixtures of both proteins. PorARc of *R. corynebacteroides*, on the other hand, contains only one negatively charged amino acid in this region of the protein that could form the periplasmic opening of the pore. This could be the reason that the pores formed by PorARc are less cation-selective than PorARr, PorBRr, and MspA (Trias and Benz 1994; Riess and Benz 2000). Besides the negatively charged amino acids (i.e., E90 and E91) at the periplasmic opening of the MspA pore exists also another conserved negatively charged amino acid in the sequence alignment shown in Fig. 2, E73. This glutamic acid is localized at the upper end of the lower β-barrel cylinder of the MspA octamer (seen from the side of the pore with the periplasmic entrance down). Since for all sequences shown in Fig. 2 the glutamic acid follows an arginine, it seems likely that its influence on channel selectivity is rather small.

### Relationship between the different subunits of the cell wall channels from the NMC-complex

**Fig. 6:**
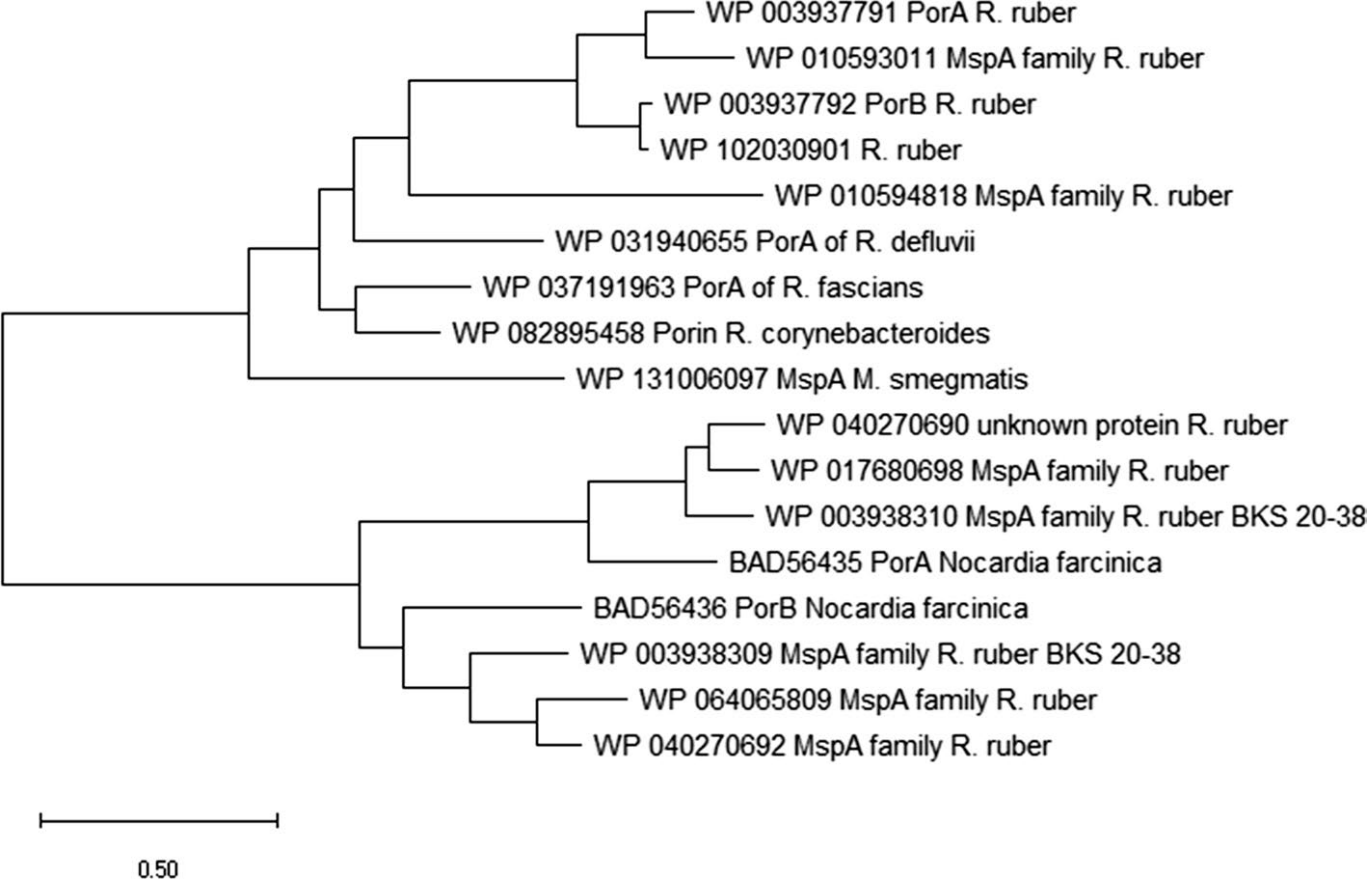
Cladogram representing the phylogenetic relationships of cell wall channel subunits of bacteria of the NMC-complex. The tree was generated using protein sequences downloaded from the NCBI protein database with the indicated identifiers. The multiple sequence alignment was calculated using Pole Bioinformatique Lyonnaise Network Protein Sequence Analysis (http://npsa-pbil.ibcp.fr). The tree was obtained using the program MEGAX (Kumar et al. 2018a)

We pointed out already that many bacteria of the NMC-complex contain cell wall channels formed by MspA-like subunits (Niederweis et al. 1999; Riess et al. 2001; Dörner et al. 2004; Kläckta et al. 2011). Protein alignment results of different subunits of cell wall channels shown in Fig. 2 suggest that these proteins are homologous. In fact, a BLAST search (https://www.ncbi.nlm.nih.gov/blast/) for homologues of PorARr and PorBRr revealed the presence of many homologous proteins in different bacteria of the NMC-complex. We constructed a phylogenetic tree of the MspA-superfamily of different subunits of cell wall channels using the program MEGAX (Kumar et al. 2018a). The results are shown in Fig. 6. The tree demonstrates that the MspA-like subunits of the different *Rhodococci* form something like their own subfamily. PorARr and PorBRr evolved presumably by gene duplication. MspA is more distantly related to the subunits from *Rhodococci*. The two subunits of *Nocardia farcinica* are only distantly related to those of *M. smegmatis* and of the *Rhodococci*. They evolved presumably also by gene duplication.

## Supplementary Information

Below is the link to the electronic supplementary material.

Supplementary file1 (DOCX 647 KB)

## Data Availability

All the data are available in the manuscript and in the Supporting Information.
