# Supplementary material for: Cell wall channels of Rhodococcus species: identification and characterization of the cell wall channels of Rhodococcus corynebacteroides and Rhodococcus ruber
Source: Eur Biophys J. 2022 May 14;51(4-5):309–23. doi: 10.1007/s00249-022-01599-9 (PMC9233653; doi:10.1007/s00249-022-01599-9)
Supplement: Supplementary file 1 — Supplementary file1 (DOCX 647 KB) [file 249_2022_1599_MOESM1_ESM.docx]

**Cell wall channels of Rhodococcus species: Identification and characterization of the cell wall channels of Rhodococcus corynebacteroides and Rhodococcus ruber**

**(Supporting Information; Mass Spectrometry Supplement)**

Claudio Piselli^1^, Lorraine Benier^1^, Cornelia Koy^2^, Michael O. Glocker^2^, Roland Benz^1^

^1^Department of Life Sciences and Chemistry, Jacobs University Bremen, 28759 Bremen, Germany

^2^Proteome Center Rostock, University Medicine Rostock, 18059 Rostock, Germany

**Mass spectrometric identification of “cell wall proteins” from SDS-PAGE bands**

To identify proteins, gel bands with cell wall proteins from *Rhodococcus corynebacteroides* and *Rhodococcus ruber* were subjected to mass spectrometric analysis of peptide mixtures which were generated by in-gel tryptic digestion. From the mass spectral data, peptide mass lists were generated and subjected to database search against an in-house curated protein sequence database, which contained all protein entries from *Rhodococcus corynebacteroides* and *Rhodococcus ruber* as well as trypsin from *Sus scrofa* plus all reviewed proteins from *Homo sapiens sapiens*. Using automatic search settings (see experimental description for details) afforded identification results (Supplemental Table S1) by “peptide mass fingerprinting”.

**Supplemental Table S1**: Identification results from “peptide mass fingerprinting” upon automated search a).

| Gel band | Protein name | Uniprot ID | No. of peptides | Sequ. cov. | Score ^b)^ |
| --- | --- | --- | --- | --- | --- |
| PorARr | MspA protein | M2ZLS3_9NOCA | 10 | 84.3 % | 8440.4 |
|  | Keratin type II | K22E_HUMAN | 14 | 21.1 % | 239.8 |
|  | Keratin type II | K2C1_HUMAN | 15 | 19.1 % | 246.4 |
|  |  |  |  |  |  |
| PorBRr | Porin | A0A098BU53_9NOCA | 2 | 11.8 % | 1655.9 |
|  | Trypsin | TRYP_PIG | 2 | 7.8 % | 305.7 |
|  | Keratin type II | K22E_HUMAN | 9 | 13.8 % | 92.6 |
|  | Keratin type II | K2C1_HUMAN | 15 | 24.1 % | 233.7 |
|  | Keratin type I | K1C10_HUMAN | 11 | 17.3 % | 231.1 |
|  | Keratin type I | K1C9_HUMAN | 6 | 10.9 % | 90.2 |
|  | Dermcidin | DCD_HUMAN | 2 | 20.0 % | 195.7 |
|  |  |  |  |  |  |
| PorARc | - | - | - | - | - |
|  | Trypsin | TRYP_PIG | 2 | 7.8 % | 206.9 |
|  | Keratin type II | K22E_HUMAN | 22 | 38.7 % | 1014.4 |
|  | Keratin type II | K2C1_HUMAN | 22 | 29.4 % | 1142.6 |
|  | Keratin type I | K1C10_HUMAN | 16 | 23.6 % | 923.0 |
|  | Keratin type I | K1C9_HUMAN | 14 | 23.0 % | 566.9 |
|  |  |  |  |  |  |

a) *Rhodococcus* proteins are shown in red; contaminations in black

b) scores below 200 are considered questionable and request manual inspection

Except for the identification results for the protein in the gel band labeled PorARr, the “peptide mass fingerprinting” identification results were not satisfactory and requested manual analysis steps to be performed, i.e. peptide mapping. Confirming with above-mentioned proteins, the following amino acid sequences were suggested to be present with the proteins of interest: PorARr (lane 1 in Figure 3 with the code WP_00397791), PorBRr (lane 3 in Figure 3 with the code WP_00397792) and PorARc (lane 4 in Figure 3 with the code WP_169818371).

Inspection of the amino acid sequence of the Msp A protein (PorARr) showed that the mature protein contained 9 tryptic cleavage sites, leaving after tryptic digestion 7 peptides which were either too long or too short, and, hence, were not separated well enough by LC chromatography, thereby escaping mass spectrometric analysis. Similarly, the mature porin (PorBRr) afforded 6 tryptic peptides, from which only 2 were within the analyzable size. The two peptides were in fact assigned to porin by automated search, but since sequence coverage was low (11.8 %), assignments needed to be verified. Routine MS^E^ analysis did not provide protein identification for the protein in gel band labeled PorARc. The assumption was that there were simply too few tryptic cleavage sites in the amino acid sequence of PorARc.

Taking the provided amino acid sequences as lead sequences allowed manual assignment of peptide ion signals from the mass spectra (see Supporting Figures S1, S2, and S3) by “peptide mapping”. Peptide ion signals served as “precursor ion signals” for following mass spectrometric fragmentation analyses. Fragment ion signals allow determinations of amino acid sequences, at least partially. In all three cases, there were only very few ion signals with high abundances in the precursor mass spectra. This was expected, as the numbers of peptides with suitable lengths was poor due to scarcity of tryptic cleavage sites.

When seeking for the masses from the mass spectra to match which with masses which were derived from the above shown amino acid sequences by performing in-silico digests and by calculating the theoretical fragment ion masses for matching these to the recorded fragment ion masses from the mass spectra, we were able to confirm the amino acid sequences of the few peptides that were produced. Despite the fact that there were only few peptide data, peptide mapping analysis provided a positive identification result in each case, confirming identification of PorARr and PorBRr from automated “peptide mass fingerprinting”.

Even for sample PorARc the assignment of the single recorded peptide ion from a *Rhodococcus* protein was possible. The PorARc amino acid sequence showed that there were only 4 cleavage sites for trypsin. From the 5 peptides which were generated by trypsin by in-gel digestion, just 1 one was accessible to MS^E^ analysis: ion signal at m/z 473.72. This ion signal is a doubly charged ion and is therefore a likely tryptic peptide candidate. Other recorded ion signals were background ion signals which came from the sample and/or from tryptic autoproteolysis. The “fragment ion spectrum” of the doubly protonated precursor ion at m/z 473.72 showed the mass spectrometrically produced fragment ions as Y″ ions. Their m/z values matched precisely to the theoretical masses, which were calculated from the amino acid sequence of the precursor ion. The singly protonated precursor ion at m/z 946.45 had survived the fragmentation procedure and was seen in the mass spectrum as well (cf. Figure S1C).

It should be noted that a routine mass spectrometric identification procedure would not accept an identification result for a protein after in-gel digestion with trypsin, which is based on just one single peptide ion and its fragment ions. One reason is that the amino acid sequence coverage is only 3.9 %. Most algorithms would be programmed to report “not convincing”, or just discard the data without reporting. However, since the mass spectrometric peptide mapping data are so clear and the precursor mass spectrum is very poor in ion signals, there is no reason to not accept this manually determined match of experimental data with the respective suggested protein sequence, hence, to confirm the presence of the respective protein in the associated gel band. The peptide mapping results are absolutely clear, and the identification results are correct.

**Figure S1**


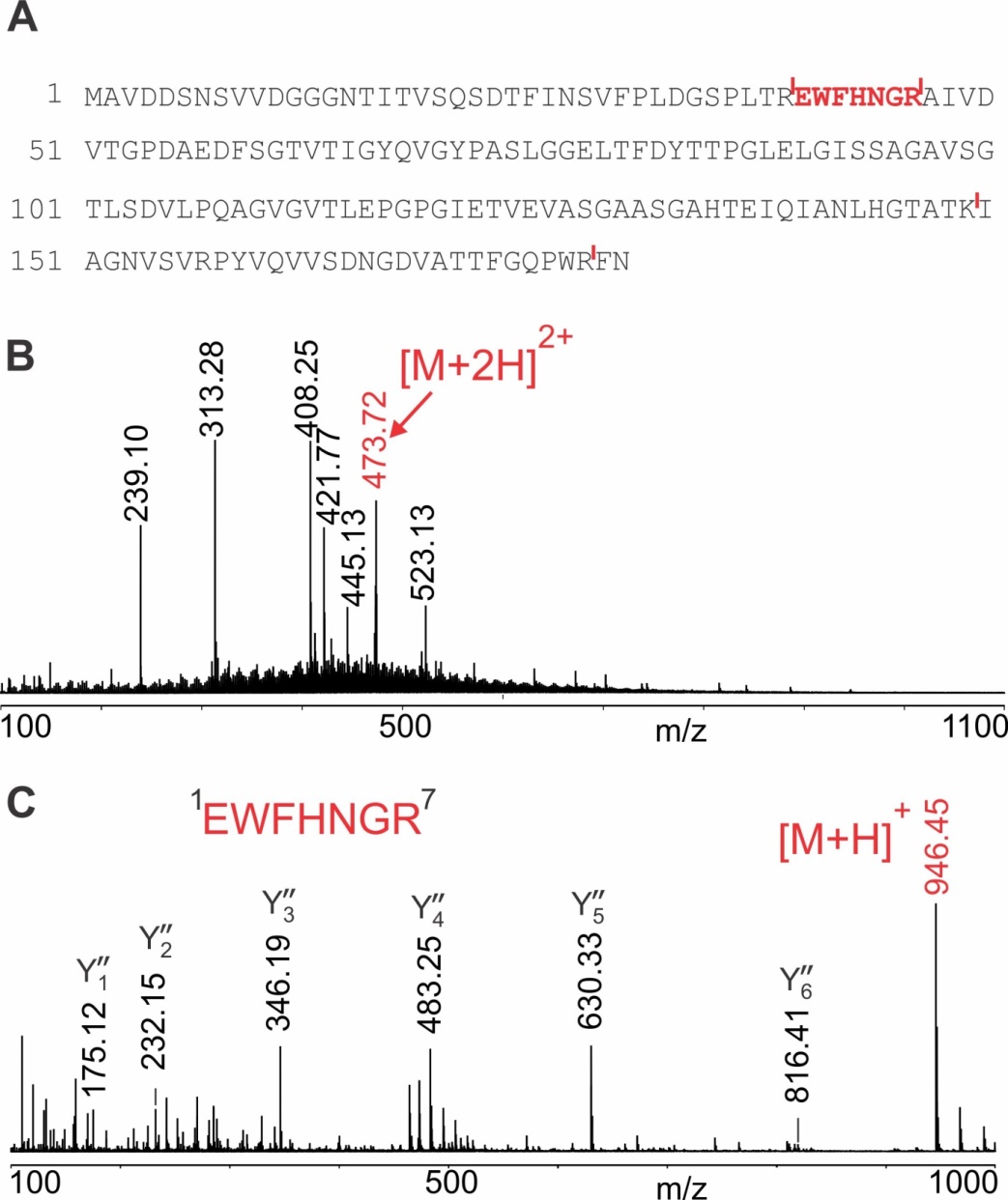


**Figure S1:** Mass spectrometric identification of PorARc. **A:** Graphical presentation of the PorARc amino acid sequence. Amino acid residues (single letter code) shown in red indicate the partial sequence which was represented by the matching ion signal in the mass spectrum. Amino acid position numberings are given at the left. **B:** Mass spectrum of the peptide mixture which eluted from the nanoLC upon tryptic in gel digestion of PorARc. The peptide ion signal which matched to the PorARc amino acid sequence is indicated by an arrow. Sequence coverage was 3.89%. The doubly protonated peptide ion signal at m/z 473.72 (precursor ion, labeled in red) was assigned to the partial amino acid sequence 40-46 from PorARc. **C:** Mass spectrum of the fragment ion mixture which was obtained by fragmentation of the precursor ion at m/z 473.72. Amino acid residues shown in red represent the partial sequence to which the Y´´n fragment ions matched, enabling positive peptide identification.

**Figure S2**


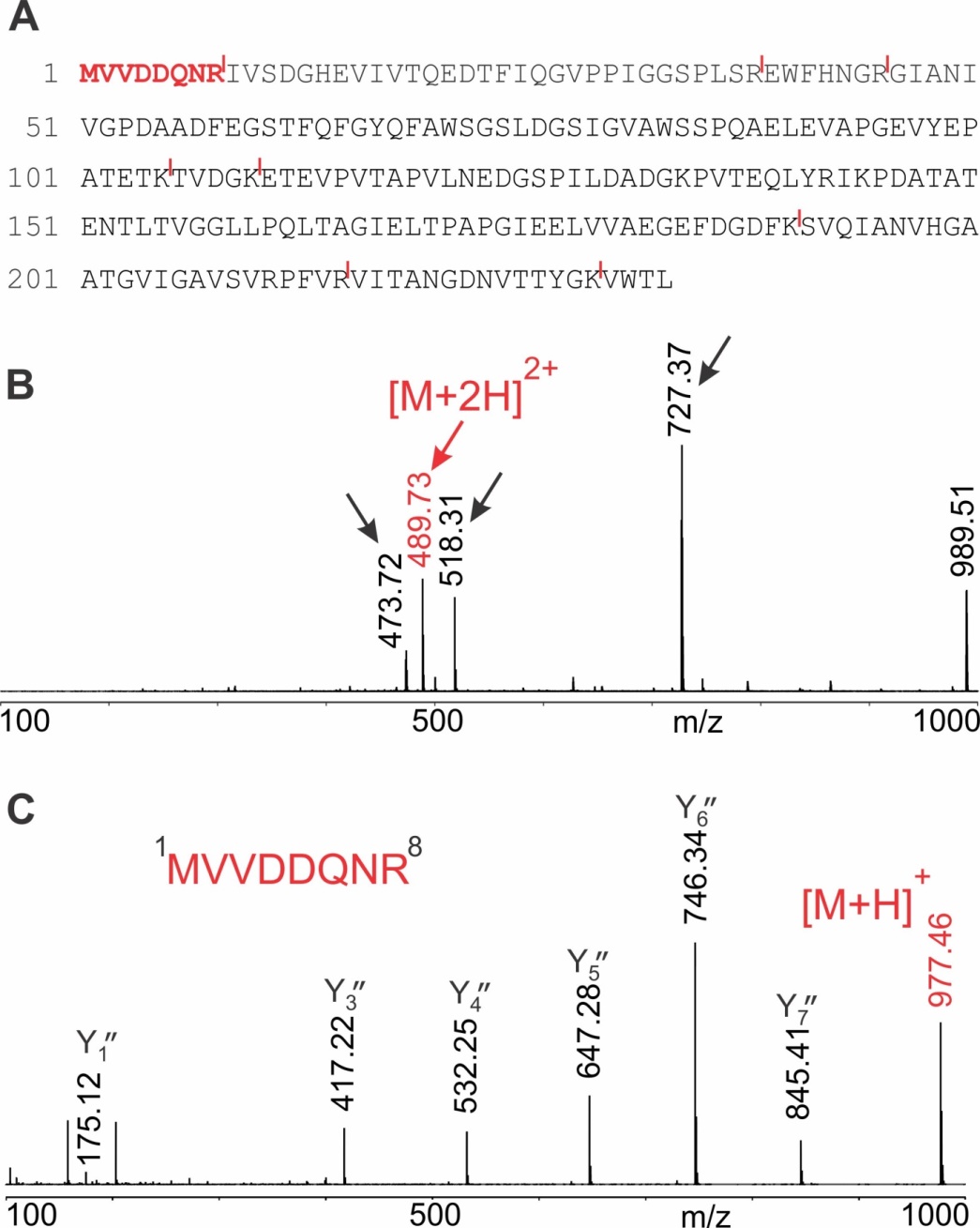


**Figure S2:** Mass spectrometric identification of PorARr. **A:** Graphical presentation of the PorARr amino acid sequence. Amino acid residues (single letter code) shown in red indicate the partial sequence which was represented by the matching ion signal in the mass spectrum. Amino acid position numberings are given at the left. **B:** Mass spectrum of the peptide mixture which eluted from the nanoLC upon tryptic in gel digestion of PorARr. The peptide ion signals which matched to the PorARr amino acid sequence are indicated by arrows. Sequence coverage was 14.16%. The doubly protonated peptide ion signal at m/z 489.73 (precursor ion, labeled in red) was assigned to the partial amino acid sequence 1-8 (N-terminus) from PorARr. **C:** Mass spectrum of the fragment ion mixture which was obtained by fragmentation of the precursor ion at m/z 489.73. Amino acid residues shown in red represent the partial sequence to which the Y´´n fragment ions matched, enabling positive peptide identification.

**Figure S3**


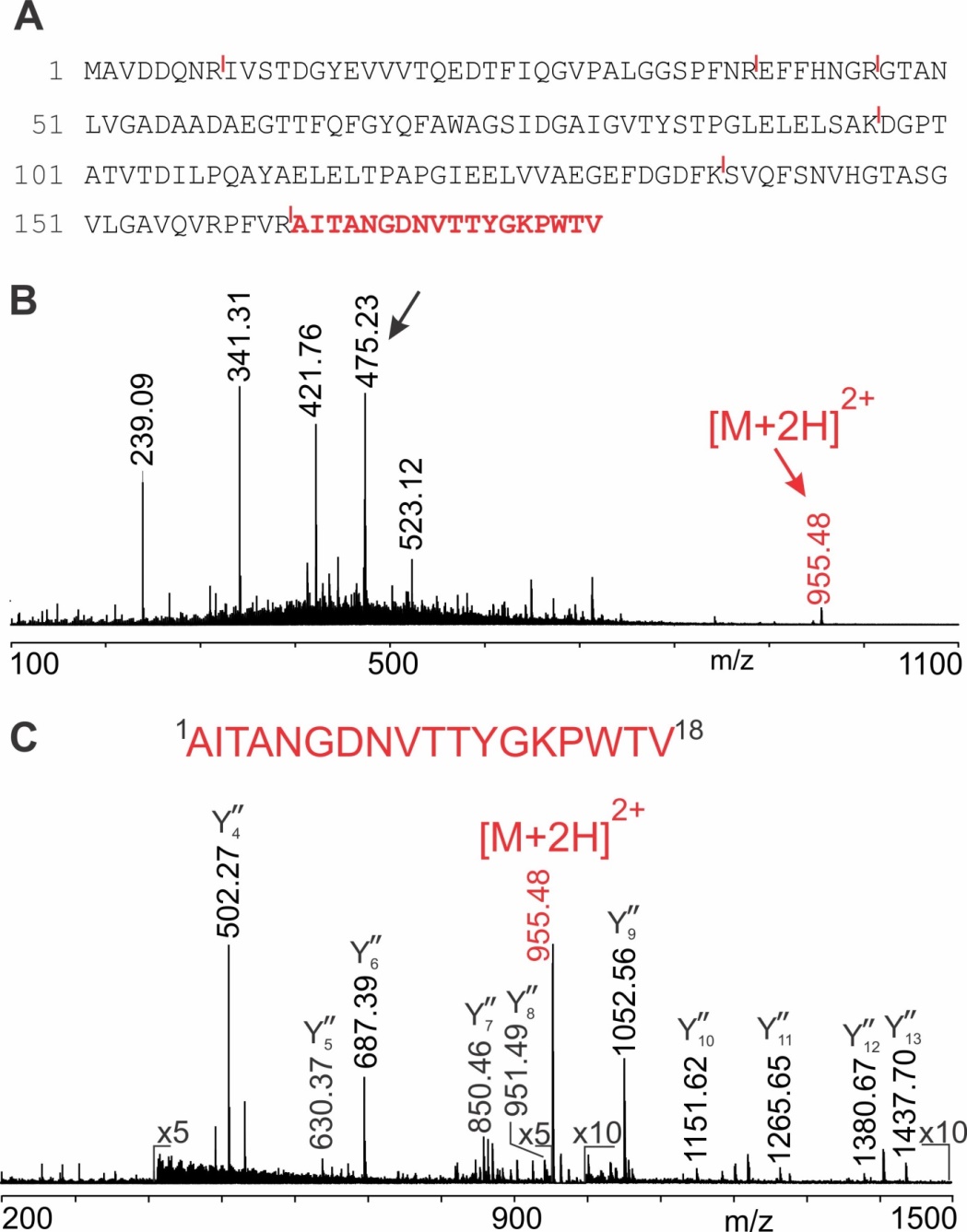


**Figure S3:** Mass spectrometric identification of PorBRr. **A:** Graphical presentation of the PorBRr amino acid sequence. Amino acid residues (single letter code) shown in red indicate the partial sequence which was represented by the matching ion signal in the mass spectrum. Amino acid position numberings are given at the left. **B:** Mass spectrum of the peptide mixture which eluted from the nanoLC upon tryptic in gel digestion of PorBRr. The peptide ion signals which matched to the PorBRr amino acid sequence are indicated by arrows. Sequence coverage was 14.44%. The doubly protonated peptide ion signal at m/z 955.48 (precursor ion, labeled in red) was assigned to the partial amino acid sequence 163-180 (C-terminus) from PorBRr. **C:** Mass spectrum of the fragment ion mixture which was obtained by fragmentation of the precursor ion at m/z 955.48. Amino acid residues shown in red represent the partial sequence to which the Y´´n fragment ions matched, enabling positive peptide identification.
